# Psychological Effects of Vision Loss and Cultural Adaptations of Resilience Building Interventions

**DOI:** 10.1093/geroni/igaf122.1740

**Published:** 2025-12-31

**Authors:** Silvia Sörensen

**Affiliations:** University of Rochester Warner School of Education and Human Development, Rochester, New York, United States

## Abstract

Affecting an estimated 19.8 million Americans aged 40 and older (about 12.6% of the population), late life vision impairment (VI) presents significant challenges to everyday functioning, social interactions, engaging in valued activities, and self-care. Depression occurs in about 1/3 of adults who develop VI. Although Latinos will constitute almost 21% of the US adult population by 2050, they are less likely than other racial/ethnic groups to access new or available treatments and interventions and are thus underserved regarding vision care and vision research. Supportive Interventions for people with VI exist in English but are not available in Spanish; many Latinos are not comfortable discussing health matters in English, so they remain undertreated. In the past 18 months we have translated our 10-week evidence-based Preventive Problem Solving Program for older adults with vision loss into Spanish and have worked with an Advisory Board of older adults to incorporate Latino cultural norms. We are currently testing the adapted intervention called “Mejorar Su Bienestar” with 8 community dwelling older Latinos who will complete all stages of the program and provide feedback. Next steps will be to build community capacity for designing and implementing a clinical trial with this community. In this presentation I will describe the psychological effects of late life VI, how interventions can address the emotional effects of vision loss, and the steps we are taking in adapting the intervention to Latino older adults in our community, with the goal to develop a national model of engagement.

